# Wing scale ultrastructure underlying convergent and divergent iridescent colours in mimetic *Heliconius* butterflies

**DOI:** 10.1098/rsif.2017.0948

**Published:** 2018-04-18

**Authors:** Andrew J. Parnell, James E. Bradford, Emma V. Curran, Adam L. Washington, Gracie Adams, Melanie N. Brien, Stephanie L. Burg, Carlos Morochz, J. Patrick A. Fairclough, Pete Vukusic, Simon J. Martin, Scott Doak, Nicola J. Nadeau

**Affiliations:** 1 Department of Physics and Astronomy, University of Sheffield, Hicks Building, Hounsfield Road, Sheffield S3 7RH, UK; 2 Department of Animal and Plant Sciences, University of Sheffield, Alfred Denny Building, Western bank, Sheffield S10 2TN, UK; 3 Department of Mechanical Engineering, University of Sheffield, Sheffield S3 7HQ, UK; 4 Mashpi Reserve, Quito, Ecuador; 5 Department of Physics and Astronomy, University of Exeter, Stocker Road, Exeter EX4 4QL, UK; 6 Department of Materials, Loughborough University, Loughborough LE11 3TU, UK

**Keywords:** structural colour, biophotonics, butterflies, iridescence, *Heliconius*, mimicry

## Abstract

Iridescence is an optical phenomenon whereby colour changes with the illumination and viewing angle. It can be produced by thin film interference or diffraction. Iridescent optical structures are fairly common in nature, but relatively little is known about their production or evolution. Here we describe the structures responsible for producing blue-green iridescent colour in *Heliconius* butterflies. Overall the wing scale structures of iridescent and non-iridescent *Heliconius* species are very similar, both having longitudinal ridges joined by cross-ribs. However, iridescent scales have ridges composed of layered lamellae, which act as multilayer reflectors. Differences in brightness between species can be explained by the extent of overlap of the lamellae and their curvature as well as the density of ridges on the scale. *Heliconius* are well known for their Müllerian mimicry. We find that iridescent structural colour is not closely matched between co-mimetic species. Differences appear less pronounced in models of *Heliconius* vision than models of avian vision, suggesting that they are not driven by selection to avoid heterospecific courtship by co-mimics. Ridge profiles appear to evolve relatively slowly, being similar between closely related taxa, while ridge density evolves faster and is similar between distantly related co-mimics.

## 1. Introduction

**Figure 1.**
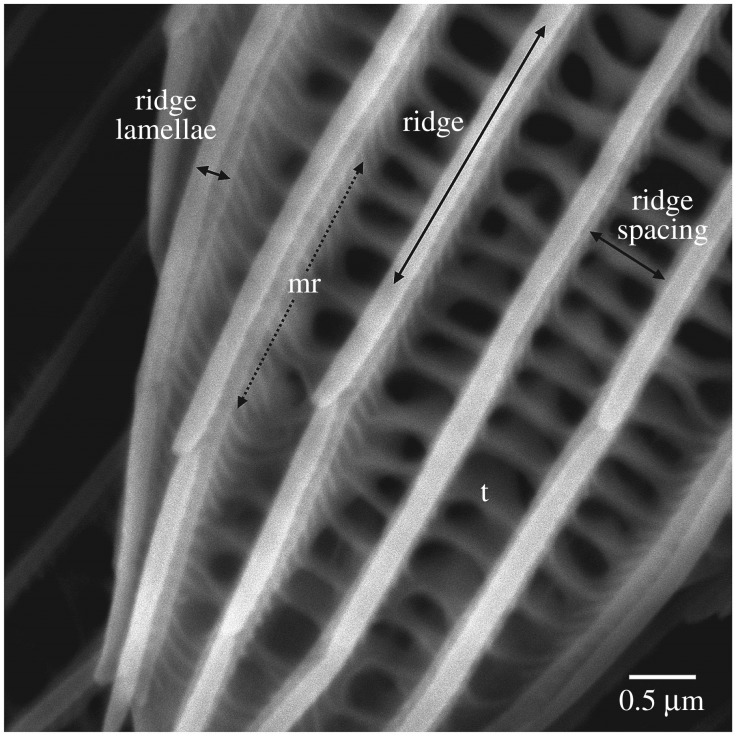
The general structures seen on a *Heliconius* wing scale. The dominant structures are the ridges, these in turn comprise the ridge lamellae, which can be overlapping as is seen in this instance. Micro ribs (mr) are found on the walls of the ridges, some continue as cross ribs, connecting the ridges together. The trabeculae (t) act as connections to the lower lamina. Image is of the tip region of a blue *H. eleuchia* wing scale, but these broad features were found on all scales.

Structural colour and in particular iridescent structural colour is widely distributed in nature, for example, in beetle elytra, the shells of molluscs, bird feathers, plants and butterfly wings [1]. It most often arises from one of two phenomena, diffraction or thin-film interference. In most butterflies studied to date, structural colours are produced by thin-film interference [2–4], when light is reflected off the upper and lower surfaces of thin films of material, in this case cuticle, composed of chitin [5], with a different refractive index (*n* = 1.56) from that of the surrounding medium (air). This creates an optical path difference between scattered light waves that gives rise to interference. The reflected colour will depend on the angle of incidence and thickness of the air and chitin layers. On butterfly wings these thin cuticle layers are present within flat scales around 100 µm in size, arranged in rows of, usually, two different forms of scale: cover scales overlapping ground scales. The surface of each scale comprises a lattice of raised longitudinal ridges joined together by cross-ribs (figure 1) [2]. The longitudinal ridges can be composed of layered lamellae, which are responsible for producing iridescence in some butterflies [3,6,7]. Little is known about the developmental processes that give rise to layered thin film structures in butterflies [7]. By comparing closely related species that differ in their structural colour we can identify how aspects of scale structure and development have evolved to optimize the reflected colour appearance [8]. An improved understanding of scale structure development could provide useful insights for replicating biological nanostructures for commercial or technological applications [9].

The neotropical *Heliconius* butterflies have been widely studied over the last 150 years, largely because of their intriguing diversity in colour and pattern and the near perfect mimicry between closely related species [10]. These studies have demonstrated that *Heliconius* colour patterns are under strong positive frequency-dependent selection due to predator avoidance of local warning patterns [11,12], which also drives mimicry between species. Colour patterns also have an important role in mate choice and mate recognition in these species, with changes in colour pattern resulting in divergent mate preference and also playing a role in driving speciation in this system [13]. The diversity of colour and pattern in this group also makes them an ideal system for understanding the mechanisms and genetic pathways controlling colour and pattern production [14,15]. The culmination of many years of genetic work has been the identification of a small number of genes that, between them, explain most of the diversity in pigmentation patterning both within and between species [16–19].

**Figure 2.**
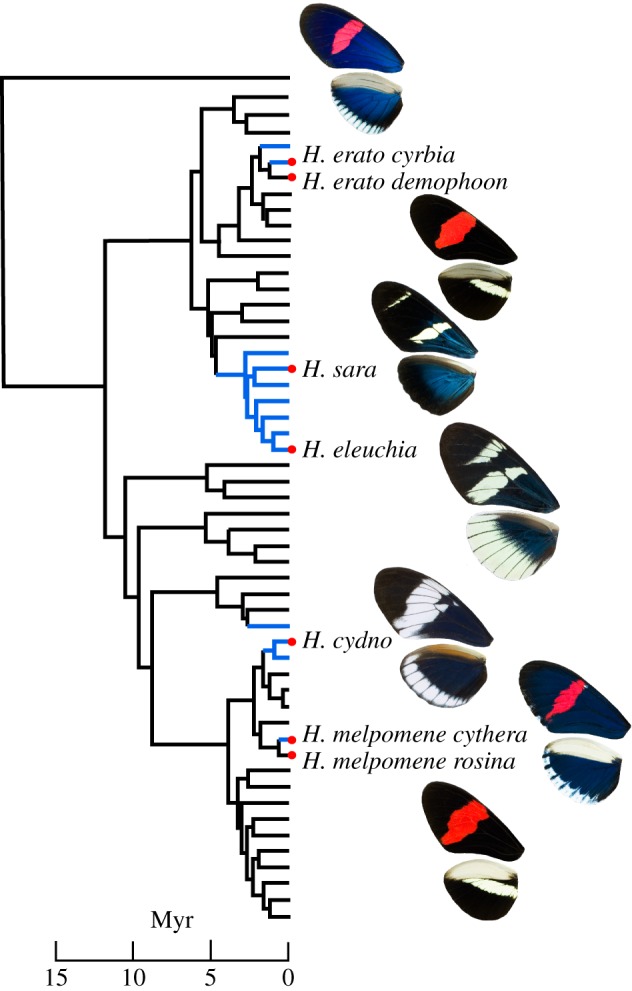
Phylogenetic relationships of the species and subspecies investigated in this work (based on [23]). Blue branches indicate the inferred presence of iridescent blue colour. Note the mimicry between distantly related species pairs *H. cydno* and *H. eleuchia*, and between *H. melpomene* and *H. erato*. Wing photographs were taken with consistent lighting conditions and camera settings. Here we focus on the blue/black wing regions only.

By contrast, very little is known about the production of structural colours in *Heliconius*. Several species exhibit an angle-dependent iridescent blue or green colour that is also mimetic between species [20,21] and involved in mate choice [22]. These colours are relatively uncommon within the greater than 40 species of *Heliconius* and appear to have evolved multiple times [23]. Iridescent blue colour is found in all members of one monophyletic group of seven species, the ‘iridescent specialists' (*Heliconius antiochus, H. leuchadia, H. sara, H. hewitsoni, H. sapho, H. congener* and *H. eleuchia*), suggesting that it likely evolved in the common ancestor of this group between 2 and 5 million years ago (figure 2) [23,24]. Similar colours in other *Heliconius* species likely have a more recent origin. For example, *H. cydno* has an iridescent blue colour, which is largely absent from its sister species (*H. timareta* and *H. melpomene*). This suggests it evolved recently in this species (fewer than 1 million years ago), likely as a result of the co-mimicry between *H. cydno* and *H. sapho* or *H. eleuchia*. Furthermore, subspecies of the co-mimics *H. erato* and *H. melpomene* from the western slopes of the Andes in Colombia and Ecuador both have an iridescent blue colour, which is absent from all other populations of these species that are widespread across South and Central America, suggesting a very recent origin (probably within the last 100 000 years [22,23]). The differences in structural colour between subspecies of *H. erato/melpomene* are genetically determined and not plastic responses to environmental differences, as they are maintained when the butterflies are reared under common conditions in captivity and hybrids between subspecies are intermediate in colour [25].

Here we characterize the structures responsible for producing iridescence in five subspecies, *Heliconius erato cyrbia* (Godart, 1819)*, Heliconius sara sprucei* Bates, 1864*, Heliconius eleuchia primularis* Butler, 1869*, Heliconius cydno alithea* (Hewitson, 1869) and *Heliconius melpomene cythera* (Hewitson, 1869), likely corresponding to four independent evolutionary origins of this type of colour. The sampled individuals were from western Ecuador where there are mimetic relationships between *H. cydno alithea* and *H. eleuchia primularis* and between *H. erato cyrbia* and *H. melpomene cythera*. We also investigated the non-iridescent subspecies *Heliconius erato demophoon* Ménétriés, 1855 and *Heliconius melpomene rosina* (Boisduval, 1870) from Panama, which are the most closely related non-iridescent populations of *H. erato* and *H. melpomene* [26], to identify the structural transitions that have occurred and give rise to iridescence in these two species. The apparent multiple recent evolutionary origins of iridescence make this group ideal to address the question of how iridescent colours evolve. In addition, the presence of mimicry between species allows us to ask how easily such colours can evolve towards a single evolutionary optimum structure or if there are subtle differences in architecture that give similar iridescent optical effects.

## 2. Material and methods

### 2.1. Butterfly specimens

Specimens of the iridescent butterflies, *H. erato cyrbia, H. sara, H. eleuchia, H. cydno alithea* and *H. melpomene cythera,* were collected in and around the Mashpi reserve in Ecuador (0.17° N, 78.87° W) between May 2014 and February 2017. One *Heliconius sara sara* (Fabricius, 1793) individual was also collected from Gamboa, Panama (9.12° N, 79.70° W), along with specimens of the non-iridescent *H. melpomene rosina* and *H. erato demophoon*. Species and subspecies identification was based on Brown [27] and Warren *et al*. [28]. For comparison, we also obtained a species with a known lower lamina reflector, the peacock butterfly (*Aglais io*), obtained from the company World of Butterflies and Moths (UK). All remaining specimens are preserved at the University of Sheffield.

### 2.2. Optical microscopy

We obtained images using a Nikon Eclipse ME600 optical microscope, using 20× or 50× objective lenses and a PixeLINK PL-A742 camera. A calibration grid (Reichert) was used to calibrate the length scales in the images. For the peacock butterfly, we used a mercury vapour lamp to give sufficient intensity in the UV/blue part of the spectrum.

We also obtained images of the *Heliconius* species using a Zeiss Axioscope optical microscope with a 100× objective lens and mounted AxioCam MR5. AxioVision software was used to obtain extended focus images by integrating information taken from images at multiple focal planes. We imaged the surface of *H. erato cyrbia* further using a Zeta-20 Optical Profiler, which characterizes the colour and the three-dimensional surface structure.

### 2.3. Scanning electron microscopy

Scanning electron microscopy (SEM) samples were prepared by cutting small regions of the wings and adhering them to SEM stubs using conductive silver paint (AGAR, UK). These were coated with a few nanometres of gold (AGAR) using vacuum evaporation and imaged on a JEOL JSM-6010LA together with InTouchScope software.

We made cross sections through the vertical ridge profile of *H. erato demophoon* and *H. sara,* as representative non-iridescent and iridescent *Heliconius* structures, respectively. The samples were sputter coated with a few nanometres of gold–palladium prior to mounting in a FEI Nova 600 Nanolab dual-beam focused ion beam (FIB) and SEM. Initial studies on these samples showed marked charging effects so individual scales were removed by bonding them to the lift-out needle of the FIB and transferring them to a clear section of the stubs. A protective layer of platinum was deposited by evaporation on the surface of each scale using FIB-induced deposition. The scales were then sectioned using a gallium ion beam and imaged using the SEM column of the system.

### 2.4. Raman microscopy

To assess the pigment content of the scales, we performed Raman microscopy on single cover scales, using a Renishaw inVia measurement system (Renishaw, UK). The butterfly scales were brought into focus using the white light source and then fine-adjusted to optimize the signal counts for the focus using the argon-ion laser (514.5 nm) source.

### 2.5. Scanning probe microscopy

A Digital Instruments Dimension 3100 scanning probe microscope (SPM) was used in atomic force microscopy (AFM) tapping mode, together with either a Nanoscope^®^ IIIa or IV controller. AFM data were taken using standard tapping mode tips (Bruker) with a resonance near 320 kHz, lowered down on either the centre or the edge of a single undamaged wing scale in the sample. The data were subsequently flattened and analysed, to produce two-dimensional surface topography height images (with height represented as a colour scale) and rendered three-dimensional surfaces using the software Gwyddion and ImageSXM. Fourier analysis was performed on the scale SPM scans and the ridge spacing extracted from the integrated Fourier transform to give an image-averaged ridge spacing over the 10 µm square image.

### 2.6. Small angle X-ray scattering

Transmission small angle X-ray scattering (SAXS) of the butterfly wings was measured at the ID02 beamline (ESRF—the European synchrotron, Grenoble, France). The camera was a Rayonix MX-170HS CCD detector and we used X-rays with a wavelength of 0.1 nm, a beam size of 20 µm by 20 µm and a sample-to-detector distance of 30.98 m. Between 8 and 17 measurements were collected over 10–20 mm from one wing of each species/subspecies.

The two-dimensional detector images were masked to account for the beam stop and edges of the detector, corrected for dark, spatial distortion, normalized by transmitted flux and subsequently radially integrated to give the one-dimensional scattering plots of scattered intensity, *I*, as a function of momentum transfer *q*, where *q* = (4πsin*θ*)/*λ*, 2*θ* being the scattering angle. Based on our interpretation of the scale structure (figure 1) and correlating this with the X-ray scattering pattern, we were able to use a particular peak position in the one-dimensional scattering pattern to measure the ridge spacing (as shown in electronic supplementary material, figure S1).

### 2.7. Optical reflectance spectroscopy

**Figure 3.**
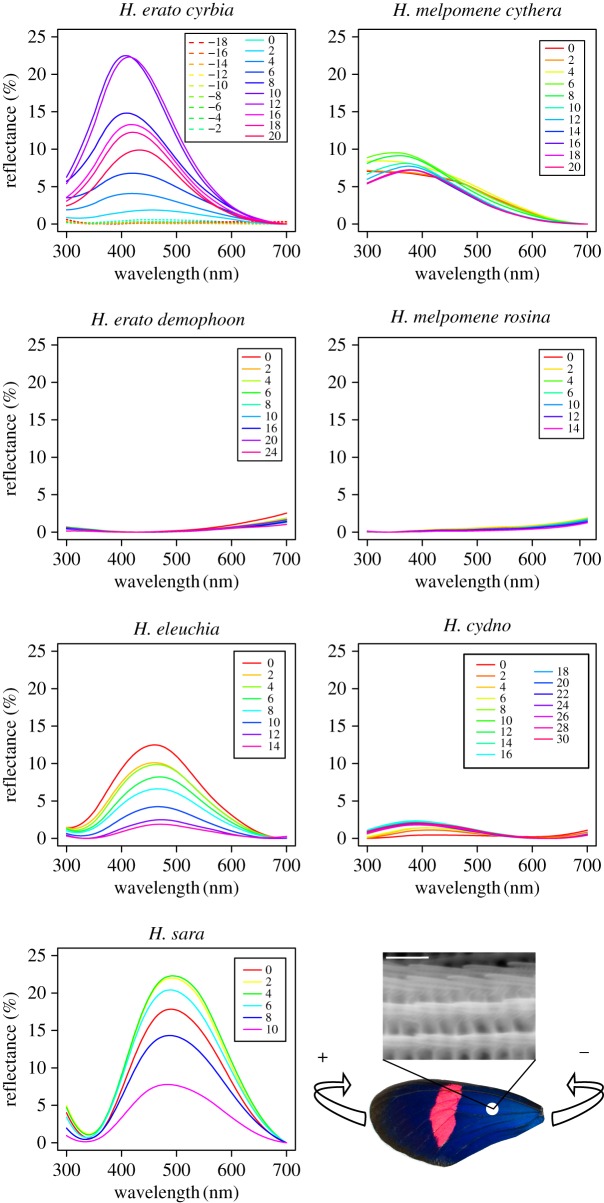
Representative reflectance profiles taken from one individual forewing of each of the seven taxa with varying angles. Angles are relative to incident with wings rotated along an anterior–posterior axis and positive angles being towards the proximal end of the wing and negative angles towards the distal end, as shown in the lower right panel. Negative angles are only shown for *H. erato cyrbia* to illustrate that the patterns are not symmetrical, due to the asymmetry of the structures when rotated along this axis.

Reflectance measurements were taken for four individuals of each taxon from both the hindwing and forewing. Samples were prepared by sticking either whole wings or pieces of wings to glass slides using a cyanoacrylate-based adhesive. The sample slide was fixed to an optical mount on a rotation stage. Measurements were taken at normal incidence and at angles parallel to the orientation of the wing scales (determined by optical microscopy as being parallel to the wing veins). Rotations were performed so that the distal part of the wing moved away from the probe, because this produced the brightest reflectance (figure 3). Measurements were taken every 2°–5° to determine the angle of brightest peak reflection for each specimen.

Reflectance spectra were captured using a spectrometer (Ocean Optics USB2000+, USA) connected through a bifurcated fibre-optic probe to a PX-2 pulsed xenon light source, the third end of the probe clamped perpendicular to the wing. Measurements were normalized to the reflectance of a diffuse white standard (polytetrafluoroethylene, Labsphere Spectralon 99% at 400–1600 nm). The SpectraSuite (Ocean Optics) software was used to collect and average 20 individual scans, with a boxcar width of 4 nm and an integration time of 1 s per scan for all sample reflectance measurements.

### 2.8. Analysis of spectral data and visual system modelling

Processing and visualization of the reflectance spectra were performed using the R package PAVO v. 1.1 [29]. The data were smoothed (using the *procspec* function with *fixneg* set to zero and *span* set to 0.3) and then normalized by subtracting the minimum reflectance of a spectrum from all wavelengths (i.e. setting the minimum to zero, using *procspec,* ‘*min*'). For some individuals, we had multiple measurements per wing, in which case we averaged these measurements using the *aggspec* function.

We used bird and butterfly visual models to compare the reflectance spectra and determine how similar the iridescent colour of different species appears to the butterflies themselves and their predators. We calculated von Kries-transformed receptor quantum catches (using *vismodel*), for two visual systems [30]. Firstly, the average avian violet sensitive model within PAVO v. 1.1, which has four photoreceptors (peak sensitivities 416, 478, 542 and 607 nm). Most avian predators of *Heliconius* are thought to have this type of visual system [21]. The model also included transmission through blackbird ocular media [31], although this had little effect on the results. Secondly, we used a *Heliconius* visual system model, which has four photoreceptors [32], with receptor sensitivities from intracellular recordings by McCulloch *et al*. [33] (peak sensitivities 355 nm—UV1, 390 nm—UV2, 470 nm—B and 555 nm—L). We first calculated relative receptor quantum catches under standard daylight for both systems, which were used for tetrahedral colour space analysis [34].

Secondly, absolute receptor quantum catches were used to calculate discriminability between each butterfly individual for forewings and hindwings separately under standard daylight (illum = ‘D65') and forest shade (illum = ‘forestshade'). We used five different visual models: avian violet sensitive, *Heliconius* type I (tetrachromatic, *H. erato* female type), *Heliconius* type II (trichomatic, *H. erato* and *H. sara* male type), *Heliconius* type III (tetrachromatic, *H. sara* female type) and *Heliconius* type IV (trichomatic, *H. melpomene* type) [35]. For the avian and *Heliconius* type I and II models we followed Finkbeiner *et al*. [36]. Specifically, for the avian visual model, we used a Weber fraction of 0.06 and relative cone abundances of VS = 0.25, S = 0.5, M = 1, L = 1. For the *Heliconius* models, we used a Weber fraction of 0.05 and relative photoreceptor abundances of UV1 = 0.09, UV2 = 0.07, B = 0.17, L = 1 for type I, and UV2 = 0.13, B = 0.2, L = 1 for type II. Type III and IV relative photoreceptor abundances were calculated from the percentages of ommatidial types multiplied by the proportion of photoreceptor types within each ommatitial type, from McCullock *et al*. [35]. For type III these were UV1 = 0.09, UV2 = 0.13, B = 0.2, L = 1 and for type IV these were UV1 = 0.07, B = 0.26, L = 1. Achromatic discriminability was based on the long wavelength photoreceptor in all cases.

### 2.9. Genotyping *Heliconius sara* individuals

To confirm the taxonomic identity of the *H. sara* individuals we sequenced a 745-bp fragment of the mitochondrial *CoI* gene, which has previously been shown to be species diagnostic for most *Heliconius* species [37], for five individuals sampled in Ecuador and one sampled in Panama (including all those used for reflectance and scale structure measurements). We used previously described protocols for polymerase chain reaction amplification with oligonucleotide primers Patlep and Jerry followed by Sanger sequencing [38]. These were then aligned with existing sequences from GenBank using BioEdit Sequence Alignment Editor v. 7.2.5. We generated a phylogenetic tree using the neighbour joining method in MEGA v. 6.06 with 1000 bootstrap replicates.

## 3. Results and discussion

### 3.1. Structural features responsible for iridescent colour in *Heliconius*

**Figure 4.**
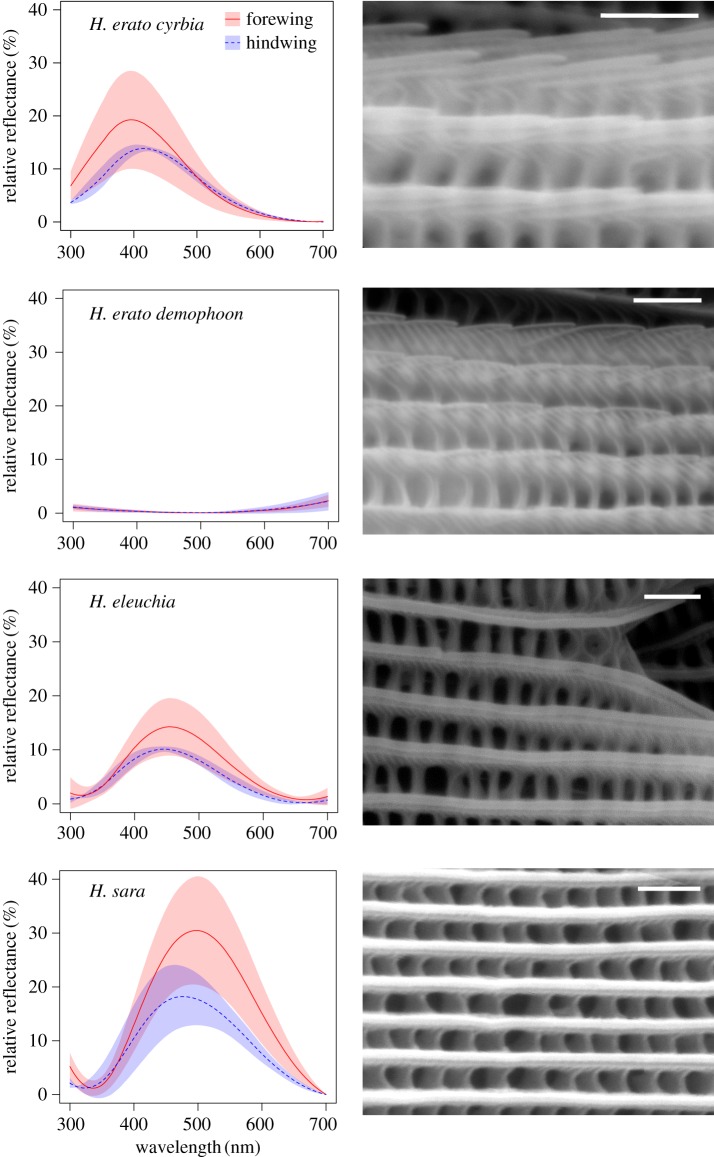
Reflectance spectra and scanning electron micrographs of wing scales for *H. erato* group species. Reflectance spectra are from the angle of maximum reflectance and are shown as the mean and standard deviation of measurements from four individuals, for both the forewing (red/solid line) and hindwing (blue/dashed line) for each species/subspecies. Scanning electron micrographs show one representative region for each species. White bars are 1 µm. (Online version in colour.)

**Figure 5.**
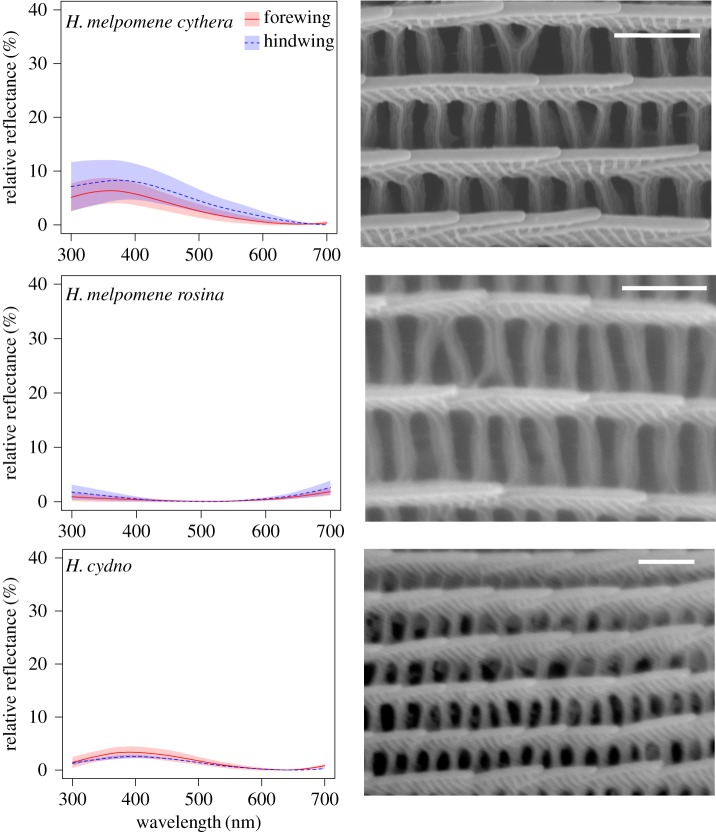
Reflectance spectra and scanning electron micrographs of wing scales for *H. melpomene* group species. See figure 4 caption for details. (Online version in colour.)

As expected, reflectance measurements taken from the blue/black region of the wings of all five species showed strong angle-dependent effects, with both brightness and wavelength of peak reflectance changing with angle (figure 3). In contrast, the ‘non-iridescent' subspecies, *H. erato demophoon* and *H. melpomene rosina,* had very little visible light reflected from their wings, which changed very little with angle. In general, reflectance from both the fore- and hindwing was similar (figures 4 and 5), suggesting that similar structures are responsible, although the forewing was generally brighter than the hindwing, perhaps suggesting some slight structural difference between the wings or possibly a difference in the density of scales on the wings or a pigmentation difference.

The general layout of an iridescent scale is shown in figure 1, with the long continuous ridges spaced by perpendicular cross-ribs. It is possible to see the trabeculae, which extend down into the scale. The ridges are structured with micro-ridges (mr) perpendicular to the length of the ridge. In the vertical axis, we see ridge lamellae. In general, the overall scale morphology was similar between iridescent blue and black scales (figures 4 and 5). However, the iridescent scales have ridge lamellae that appear to overlap in vertical layers, which is not observed in the non-iridescent taxa. It is this series of chitin/air/chitin lamellae repeat structures that we propose causes the iridescence in the *Heliconius* butterflies we have examined. This is in agreement with a recent analysis of wing scale structure in *H. sara* [39]. This type of structure has been described in other butterfly species previously [2], most notably as producing the bright blue colour of *Morpho* butterflies [3].

**Figure 6.**
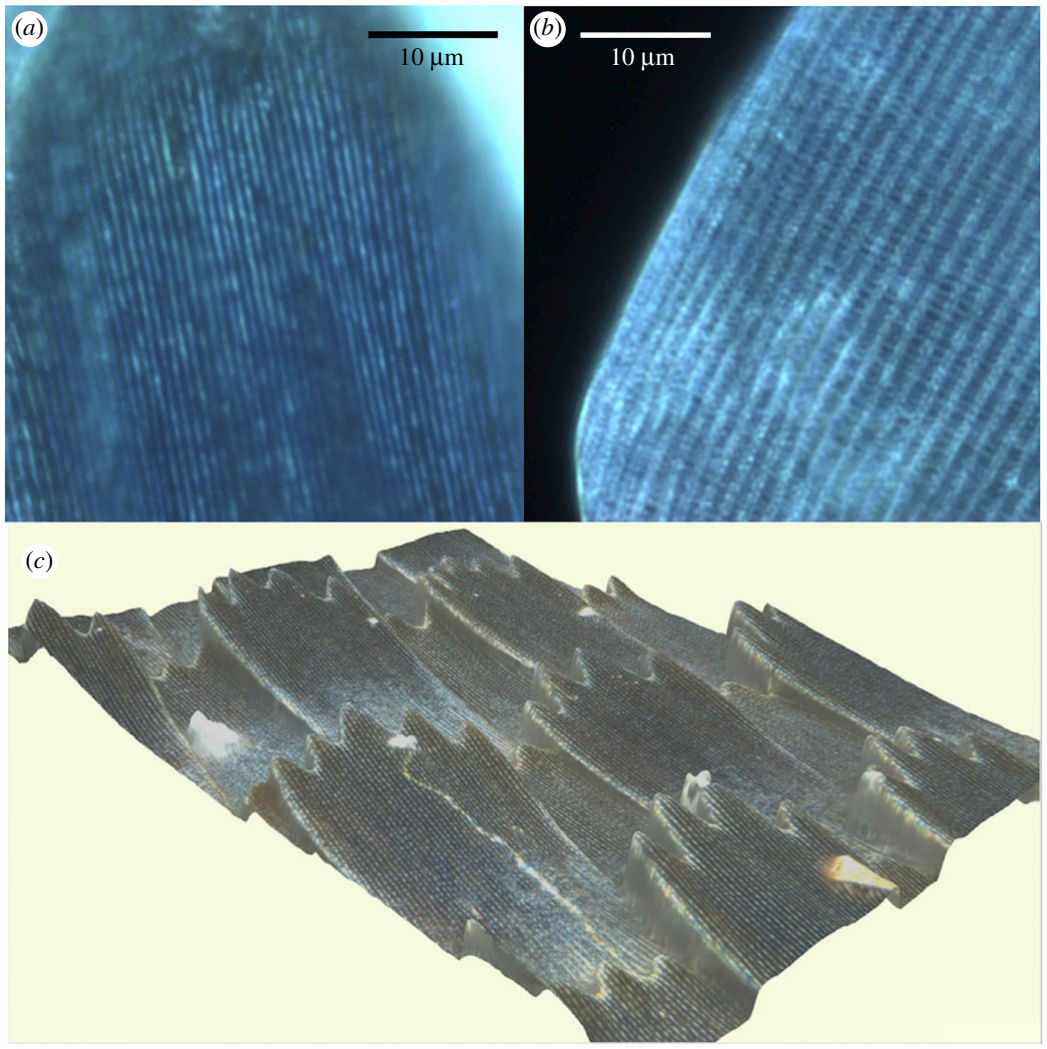
Optical microscope images showing that the blue colour localizes to the ridge structures in *Heliconius*. (*a*) Extended focus, dark field microscopy image of part of two scales on a *H. erato cyrbia* wing. (*b*) For comparison, part of two blue scales on the wing of *Aglais io*, a known lower lamina reflector. (*c*) True colour three-dimensional image from the Zeta-20 Optical Profiler, of scales on a *H. erato cyrbia* wing.

**Figure 7.**
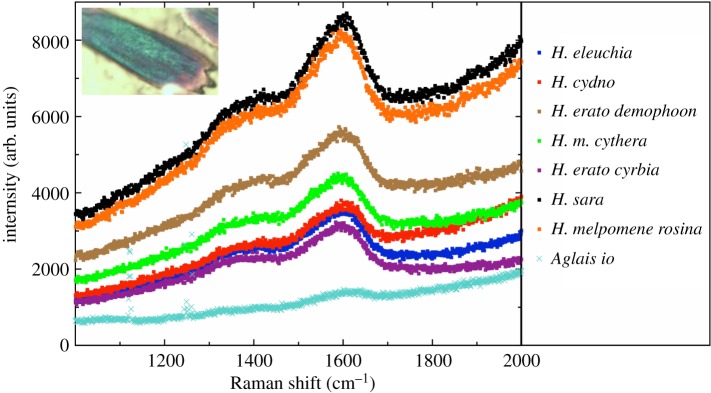
Single scale Raman spectra measurements from cover scales. The melanin peak appears at wavenumbers 1588 and 1408 cm^−1^ [41], this signal being due to the in-plane stretching of the aromatic rings and linear stretching of the C–C bonds within the rings, and is also accompanied by components from the C–H vibrations in both the methyl and methylene groups. The measured blue wing regions of *H. erato cyrbia*, *H. cydno* and *H. eleuchia* contain similar melanin concentrations to the black wing regions of *H. erato demophoon* and *H. melpomene rosina*.

Optical microscopy showed that reflected blue colour in all five *Heliconius* species was highly localized to the ridges (figure 6; electronic supplementary material, figure S2). In some other nymphalid butterflies, such as the peacock butterfly, *Aglais io*, the lower lamina of the scale has been shown to act as an optical thin film and reflect blue light [40]. Optical microscope images from the blue eye spots of the peacock butterfly were very different from those taken from the five *Heliconius* species, with reflected colour seen in many places across the scale rather than localized to the ridges, supporting our assertion that the blue colour seen in these *Heliconius* species is not due to lower lamina reflection. In addition, Raman spectra of the blue *Heliconius* cover scales show a high amount of melanin present, of a similar order to that seen in the black wing regions of the non-iridescent taxa (figure 7), which is also consistent with the fact that the iridescent wing regions appear black at certain angles (figure 3). This melanin would likely inhibit any reflection being discernible from the lower lamina; indeed, most butterfly scales that derive blue colour from the lower lamina have little or no pigment present [42].

Images of individual *H. erato cyrbia* scales show that both cover and ground scale types are similar in appearance and reflect blue colour equally (electronic supplementary material, figure S2), suggesting that layering of the scales on the wing does not have a major influence on colour production, as it does in some butterflies [3,39].

**Figure 8.**
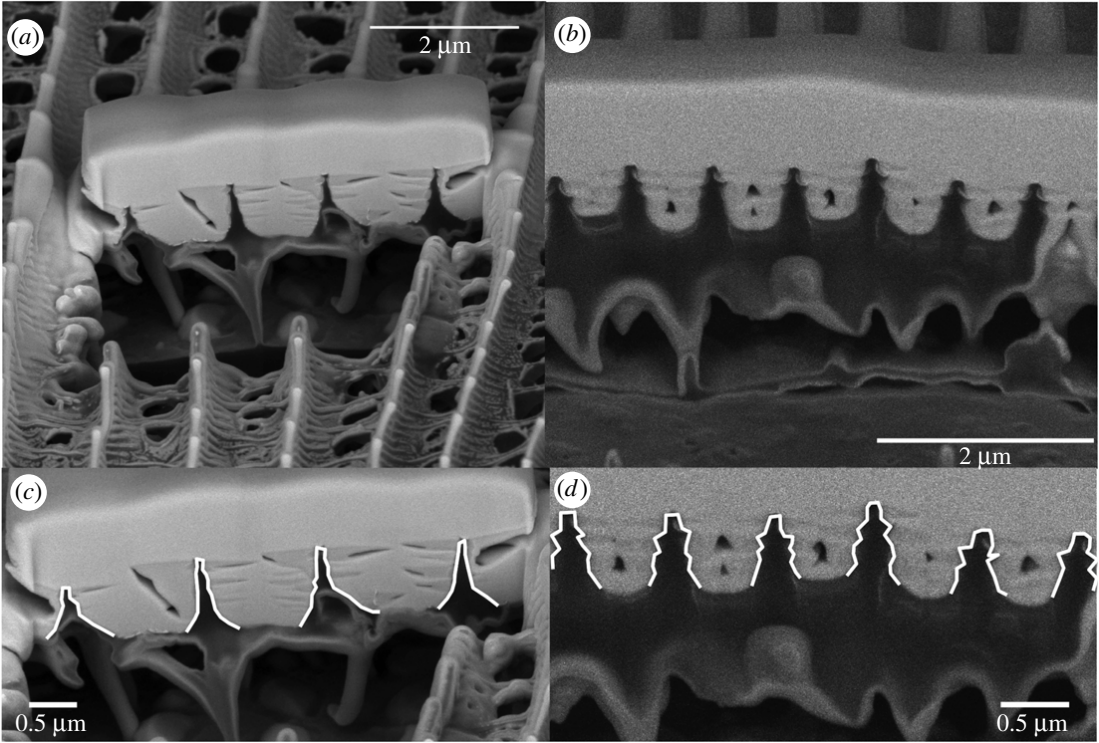
Focused ion beam interrogation of the surface of a *H. erato demophoon* cover scale (*a*) and a *H. sara* cover scale (*b*) showing crenelated like structures perpendicular to the ridge surface. In (*c*) and (*d*) the vertical ridge profile is highlighted in white to appreciate the difference between non-iridescent *H. erato demophoon* (*c*) and iridescent *H. sara* (*d*).

To examine the ridges in cross section, we used a focused ion beam to section them without the need macroscopically to cut and possibly damage the scale structure. The ridge structure of the non-iridescent *H. erato demophoon* appears non-periodic with a near-smooth triangular profile (figure 8*a,c*). In contrast, the ridge structure of bright iridescent *H. sara* showed approximately two regular periodic chitin protrusions (figure 8*b*,*d*), which we propose are responsible for the iridescence. The air space and chitin layers were 119 ± 6 nm and 85 ± 7 nm, respectively. In total, this reveals the period for the structure to be 204 ± 10 nm and yields an effective (average) refractive index of 1.22. These values can be used to give the reflected wavelength of 499 ± 18 nm [43], in good agreement with the measured reflectance peak for *H. sara* (Ecuador, figure 4 and table 1).

**Table 1.** Summary of reflectance parameters and ridge spacing measurements for all taxa.

species	ridge spacing from AFM (nm)	ridge spacing from SAXS (nm)^b^	wavelength of peak reflectance^a^ (nm)	full width at half maximum^a^ (nm)	angle of maximum reflectance (°)^a^	maximum reflectance value^a^ (%)^b^
*H. erato cyrbia*	887	812 ± 28	369 ± 8	177 ± 12	11.8 ± 4.9	19.4 ± 9.3
*H. erato demophoon*	1149	1063 ± 39	∼700	n.a.	n.a.	2.4 ± 1.4
*H. sara* (Ecuador)	723	742 ± 28	497 ± 7	191 ± 5	10.0 ± 7.2	30.6 ± 10.1
*H. sara* (Panama)	—	—	443 ± 3	274 ± 2	3.5 ± 0.7	15.9 ± 2.1
*H. eleuchia*	1159	889 ± 73	455 ± 12	172 ± 1	6.0 ± 3.5	14.4 ± 5.3
*H. cydno*	1143	929 ± 58	390 ± 2	192 ± 20	6.7 ± 3.0	3.4 ± 1.1
*H. melpomene cythera*	1242	823 ± 22	362 ± 10	180 ± 9	16.7 ± 4.6	6.4 ± 2.4
*H. melpomene rosina*	1253	1025 ± 82	∼700	n.a.	n.a.	1.9 ± 0.7

^a^Measurements are from within the bird/butterfly visible range (300–700 nm) from forewings given as mean values from four individuals ± s.d., except for *H. sara* from Panama, where only a single individual was measured and values are means of three measurements.

^b^These values are plotted in figure 10.

### 3.2. Comparison of optical and structural features between *Heliconius* species

Optical microscopy also revealed differences between iridescent *Heliconius* species. *Heliconius sara* has long near-continuous lines of colour whereas *H. erato cyrbia, H. melpomene cythera* and *H. cydno* have punctuated colour along the length of the ridges (figure 6; electronic supplementary material, figure S2). These optical differences can be explained by observed differences in ridge morphology between species. In the SEM images of *H. sara* the ridges are essentially flat and uninterrupted, while in all other taxa the ridge lamellae are more steeply sloped and hence are not continuous and appear to have a variable number of layers of lamellae long their length (figure 4). This suggests that the observed breaks in the lines of colour along the ridges are due to parts of the ridges where the lamellae either do not overlap or where the spacing of the overlapping layers is not of the correct periodicity to cause constructive interference of a visible wavelength.

**Figure 9.**
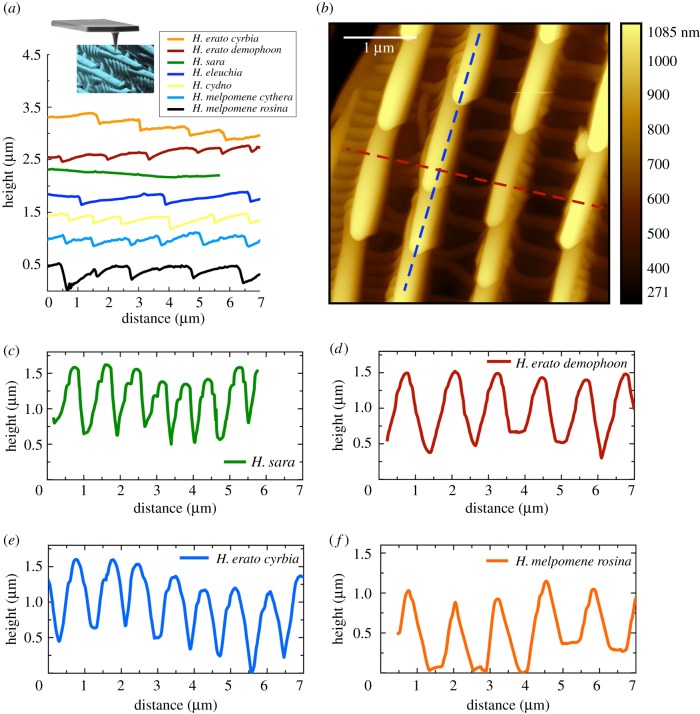
(*a*) The ridge profile extracted from a single ridge for each of the seven taxa using AFM scans. The inset shows an AFM tip scanning along a cover scale ridge structure. (*b*) A representative AFM image of the surface of a blue iridescent region for *H. cydno* showing the saw tooth like ridge discontinuities. The blue dashed line highlights the ridge profile shown in (*a*), while the orange dashed line shows the ridge heights, shown in (*c*–*f*) for two iridescent and two non-iridescent subspecies. (Online version in colour.)

There was substantial variation between individuals within some of the species for the angle at which maximum reflectance was observed (table 1). This is most likely due to the wing surfaces being uneven. Nevertheless, there were some apparent differences between species, with *H. eleuchia* and *H. sara* generally showing maximum reflectance at angles closer to normal incidence (0°) than *H. erato cyrbia* and *H. melpomene cythera*. Figure 9*a* shows ridge profiles for each of the seven taxa extracted from SPM data from wings in their natural state. For *H. sara* and *H. eleuchia* the structures were fairly flat, which likely explains why the angle of maximum reflection is close to normal incidence. *H. erato cyrbia* and *H. melpomene cythera* have more angled ridge lamellae, with the angle of peak reflectance occurring when the wing is rotated such that the beam and detector are perpendicular to these. Rotating the wing in the opposite direction produced very low reflection (figure 3), as in this direction the light beam does not fall perpendicular to the lamellae.

There were also considerable differences in brightness between the five iridescent taxa, with *H. sara* and *H. erato cyrbia* being the brightest and *H. cydno* showing the weakest reflectance (figures 4, 5 and table 1). The SPM data suggested two major features of scale morphology giving rise to this variation. Firstly, as noted above, the planar nature of the *H. sara* ridges likely contributes to the brighter overall optical effect, because colour is reflected across the entire length of the ridge, with good uniformity in the ridge optical nanostructure. The taxa with the weakest iridescent colour, *H. cydno* and *H. melpomene cythera*, have curved ridge profiles, which reduces the uniformity of the lamellae layers. In this respect, they are somewhat similar to the non-iridescent taxa, which also have highly curved ridge profiles (figure 9*a*). Secondly, ridge reflectors were much more densely packed in *H. sara* (ridge spacing at 723 nm) and *H. erato cyrbia* (887 nm) than the other species, where spacing was above 1 µm, when measured using SPM over 100 µm^2^ (table 1). The SPM data show that the ridge height is fairly consistent between taxa (figure 9*c–f*), suggesting that these species do not produce brighter colours by substantially increasing the number of layers of lamellae.

**Figure 10.**
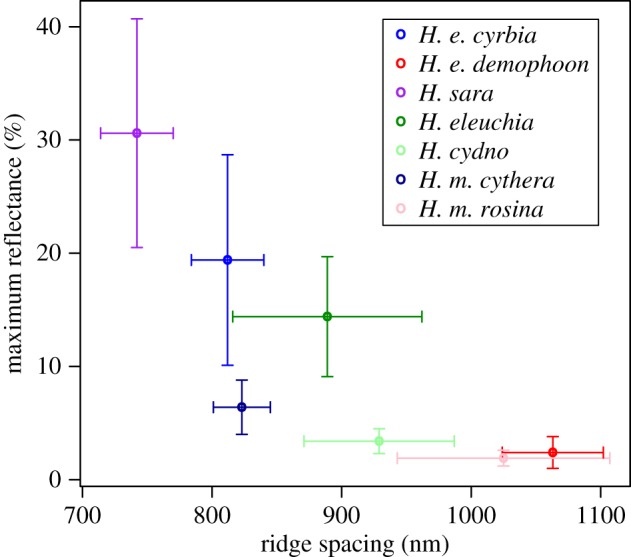
Taxa with lower ridge spacing (higher ridge density) have higher peak reflectance. The slope of the relationship is greater for taxa in the *H. erato* group (*H. e. demophoon, H. e. cyrbia, H. sara, H. eleuchia*) than for taxa in the *H. melpomene* group (*H. cydno, H. m. cythera, H. m. rosina*), likely due to differences in the structure of the ridges themselves, as observed with SEM and AFM. Co-mimetic pairs *H. e. cyrbia/ H. m. cythera* and *H. eleuchia/H. cydno* have similar ridge density. Values are mean ± s.d. for each species/subspecies, as shown in table 1. Ridge spacing is calculated from SAXS data from 8–17 measurements across one wing of each species/subspecies. Reflectance values are from four individuals of each species/subspecies.

Ridge spacing was also inferred from the SAXS patterns (electronic supplementary material, figure S1), and correlated with peak reflectance (Pearson correlation from mean values, *r* = −0.829, *p* = 0.021; table 1 and figure 10). These ridge spacing values were derived from many measurements, each an average over 400 µm^2^, so are likely to be better estimates of the average ridge spacing than the SPM measurements, which will be subject to individual scale variability. This comparison also confirmed that the *H. melpomene* clade species (*H. cydno* and *H. melpomene cythera*) achieve lower reflectance for a given ridge density than the *H. erato* clade species (*H. sara, H. eleuchia* and *H. erato cyrbia*).

There were also differences between species in the wavelength of the peak reflectance (hue), with *H. sara* being the greenest (figure 4 and table 1). These differences are presumably due to differences in the spacing of the ridge lamellae layers, although data from further cross sections of the ridge structures are needed to confirm this. Our prediction is that the spacing of the lamellae layers will be greater in *H. sara* than in the other iridescent species, leading to constructive interference of longer wavelengths.

### 3.3. Colour differences between species and visual modelling

Our measured wavelength of peak reflectance for *H. sara* was considerably longer than that reported previously for samples collected from Panama [21]. We, therefore, measured a *H. sara sara* from Panama, which had a wavelength peak similar to those measured by Thurman & Seymour [21] (electronic supplementary material, figure S3), suggesting that this is a regional difference in *H. sara*. To confirm that we were not in fact measuring two different species, we genotyped our *H. sara* individuals from Ecuador and Panama for the COI gene and compared them to existing *Heliconius* sequences for this gene. Of the five genotyped *H. sara* individuals from Ecuador, three were identical to the reference *H. sara* sequence on GenBank, and they showed between 98.3% and 98.5% identity to the individual from Panama. In addition, all sampled *H. sara* individuals grouped together robustly in a phylogenetic tree (electronic supplementary material, figure S4), strongly supporting a single species designation.

*Heliconius sara* may have shifted to being more green in appearance in this population west of the Andes in Ecuador because none of its main co-mimics (*H. leucadia*, *H. antiochus* and *H. congener* and *H. wallacei*) are present in this area [27,44]. The only mimic that is present is *H. doris*, but the blue colour in this species is not of the same type [39]. *Heliconius doris* is also polymorphic, with blue, green and red forms.

In order to better understand the selective pressures driving the evolution of these traits, we used visual models to assess and quantify the extent of similarity in colour between co-mimetic taxa. Birds are thought to be the main visual predators of *Heliconius* and as such to be the main force driving mimicry between species [10,45]. Birds are tetrachromatic, with four different photoreceptor types that allow colour discrimination along four axes. Avian visual systems can be broadly classed into two types, violet and UV sensitive, based on the peak absorbance of their shortest wavelength sensitive opsin [46,47]. The main butterfly predators in South America belong to the tyrant flycatcher (Tyrannidae) and jacamar (Galbulidae) families [45], both of which have visual systems that are sensitive to violet but not ultraviolet colours [46].

**Figure 11.**
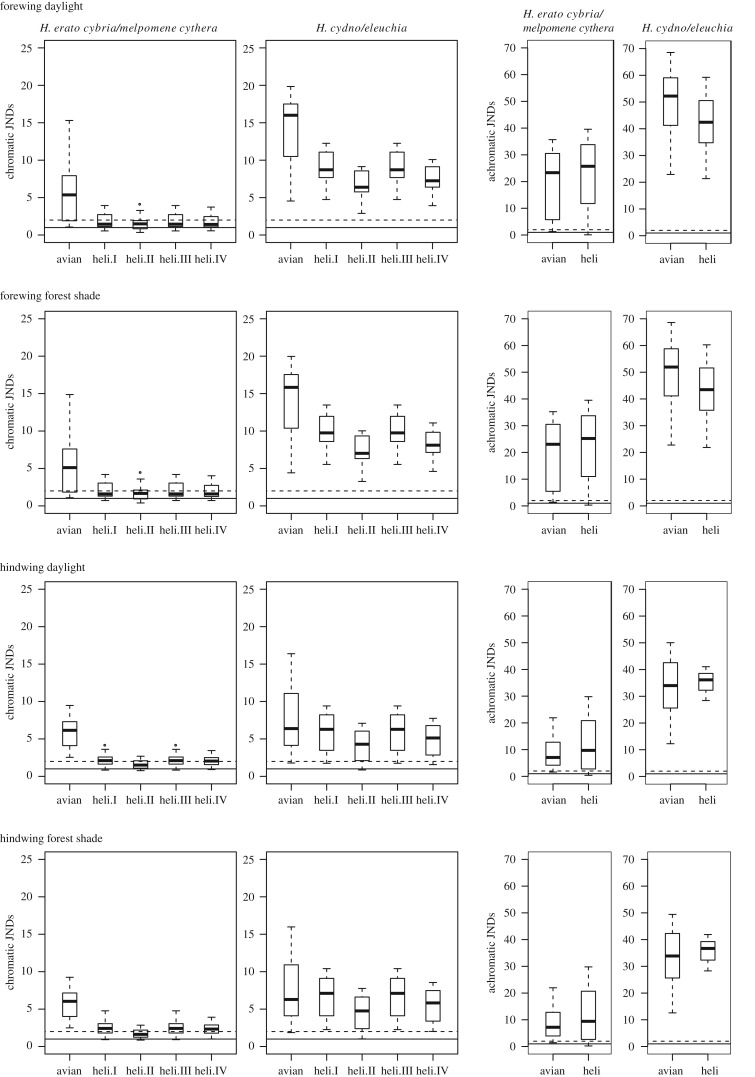
Discriminabilities of co-mimetic species pairs (*H. erato cyrbia* versus *H. melpomene cythera* and *H. cydno* versus *H. eleuchia*) for iridescent forewing and hindwing regions under ideal daylight and forest shade. The left plots give chromatic (colour) discrimination and the right plots give achromatic (brightness) discrimination. Units are just noticeable differences (JNDs), with values above 1 indicating that the colours can be discriminated (solid line threshold in plots; a threshold of 2 is also indicated with a dotted line). Five visual systems are compared: avian violet sensitive (avian), *Heliconius* type I (heli.I, tetrachromatic, *H. erato* female type), *Heliconius* type II (heli.II, trichomatic, *H. erato, H. sara* and presumed *H. eleuchia* male type), *Heliconius* type III (heli.III, tetrachromatic, *H. sara* and presumed *H. eleuchia* female type) and *Heliconius* type IV (heli.IV, trichomatic, *H. melpomene* type). Boxes indicate the upper and lower quartiles and median of all pairwise comparisons between individuals of each species (*n* = 16). Tails indicate the maximum and minimum values excluding outliers (if present, shown as points).

Wing colours are also important cues for the butterflies themselves, particularly in mate choice [10]. The visual systems of butterflies are very different from those of birds [33]. In particular, the *Heliconius* butterflies have undergone a duplication of the UV sensitive opsin, allowing them to see additional colours in the UV range [32]. This raises the possibility that some differences in colour between co-mimics may be visible to the butterflies but not avian predators. This would presumably be a selective advantage, allowing the butterflies to distinguish conspecifics from mimics, and so prevent wasted effort courting non-conspecifics, while gaining the protection benefits of mimicry. We would, therefore, predict co-mimics to be less discriminable in models of bird vision when compared to butterfly vision. However, the results of visual modelling found the opposite to be the case (figure 11). Under both ideal daylight and forest shade lighting and for both forewings and hindwings, co-mimetic species were more discriminable with the avian visual model than any of the *Heliconius* models. Achromatic discriminabilites were similar for the birds and butterflies, but very high for both taxa, suggesting that both visual systems could easily discriminate species based on brightness. This suggests that the lack of perfect mimicry between species is not due to selection for conspecific recognition, and instead may be due to developmental constraint in the ability to rapidly evolve specific photonic structures.

This result is surprising, given that the *Heliconius* species largely differ along the blue–UV axis. This can be seen in a colour space analysis, where the species do appear more distinct in *Heliconius* visual space than in avian visual space (electronic supplementary material, figure S5). The reason the discriminability analysis finds poorer discrimination with the *Heliconius* visual systems is that it takes into account relative photoreceptor densities: the *Heliconius* visual system has relatively fewer photoreceptors sensitive in the blue/UV range, even though it has more types within this range. Nevertheless, the visual models are a simplification of the visual systems and little is known about precisely how the relative abundances and distribution of photoreceptor types will influence colour discrimination [48,49].

Recent work by McCulloch *et al*. [35] has shown that a change occurred in the visual system on the branch leading to the ‘iridescent specialists' including *H. sara* and *H. eleuchia*, suggesting that the evolution of iridescence and the change of the visual system occurred around the same time. This change only occurred in the female visual systems: the males of both *H. erato* and *H. sara* do not express the UV1 opsin (type II visual system), while females of both species do (type I and type III visual systems in *H. erato* and *H. sara* females respectively) [35]. In the visual models, the male type II system is less able to discriminate the co-mimics (figure 11; electronic supplementary material, figure S5), suggesting that iridescent colour may have a more important role in female than male mate choice. In the *H. melpomene* clade, both sexes have a trichromatic visual system, lacking the UV2 opsin (type IV visual system) [35], but their predicted discrimination ability is better than the type II visual systems, suggesting that the UV1 opsin is more useful for discriminating these colours. We also note that the discrimination ability of the *Heliconius* visual systems is slightly better under forest shade lighting conditions than under standard daylight, while the opposite is true of the avian visual system (figure 11), which may suggest an advantage to these types of colours in more shaded environments.

There are other aspects of these iridescent signals that are not captured by visual modelling. Firstly, both the brightness and wavelength of peak reflectance change with angle (figure 3). This may mean that differences that are obvious to predators when measured on stationary wings may not be perceived as such on live, moving individuals. Secondly, the layered thin film reflectors that produce the iridescent colour are also known to produce polarization of the reflected light [22,24,49,50]. This can be detected by butterflies [22,51,52], but probably not by avian predators. Therefore, the differences in the ridge reflectors that we have documented between species could allow species discrimination by producing different polarization signals, even though the colours seem less readily discriminated by the butterflies than avian predators.

### 3.4. Evolutionary insights into the biological process of constructing a ridge reflector

Within *Heliconius* it appears that modification of the scale ridges to produce multilayer reflectors has occurred multiple times. Nevertheless, co-mimetic species do not appear to have achieved perfect mimicry in these structural colours, suggesting that there are developmental constraints in the evolutionary ability to modify the ridge reflectors. The brightest iridescence and highest degree of modification of the scale ridges is seen in *H. sara*, which belongs to a clade of all iridescent species, which likely evolved iridescence several million years ago. This suggests that structural changes have accumulated gradually over evolutionary time to produce brighter structural colour in this group. In contrast, *H. erato* is remarkable in that it appears to have very recently evolved relatively bright structural colour.

There appear to be three key features that determine the variation in hue and brightness of structural colour in these species: ridge density, curvature of the lamellae that make up the ridges and layering of the lamellae. Ridge density appears to evolve relatively rapidly, with mimetic species having similar ridge density (table 1 and figure 10). In contrast, ridge curvature appears to be more slowly evolving in these species, with distinct differences observed between the two major clades: the *H. erato* clade species (*H. erato, H. eleuchia* and *H. sara*) all appear to have flatter ridge profiles, while the *H. melpomene* clade species (*H. melpomene* and *H. cydno*) have more curved ridge profiles (figure 9). This also holds for the non-iridescent taxa: *H. erato demophoon* appears to have a less curved ridge profile than *H. melpomene rosina*. This may explain why *H. erato cyrbia* was able to rapidly evolve bright iridescent structural colour, while *H. melpomene cythera* and *H. cydno* appear not to have been able to.

Previous work on other butterflies has shown that the ridges form between longitudinal actin filaments during development [54–56]. Less is known about what causes folding of the ridges in order to produce the ridge lamellae. Ghiradella [56,57] has proposed that they could be produced by buckling of the ridges under mechanical stress and that the actin filaments may also be responsible for producing this stress. This would be consistent with results from Dinwiddie *et al.* [54] who showed that actin also played a critical role in the elongation of the cell. The *Heliconius* butterflies provide an ideal system to understand these processes better through comparisons of scale development and molecular genetics.

## Supplementary Material

Supplementary Figures

## Supplementary Material

Associated data

## Data accessibility

The datasets supporting this article have been uploaded as part of the electronic supplementary material.
