# Supplementary material for: Wing scale ultrastructure underlying convergent and divergent iridescent colours in mimetic Heliconius butterflies
Source: J R Soc Interface. 2018 Apr 18;15(141):20170948. doi: 10.1098/rsif.2017.0948 (PMC5938584; doi:10.1098/rsif.2017.0948)
Supplement: Supplementary Figures [file rsif20170948supp1.pdf]

Supplementary Information for

Parnell, A.J., Bradford, J.E., Curran, E., Washington, A.L., Adams, G., Brien, M.N., Burg, S.L., Morochz, C., Fairclough, J.P.A., Vukusic, P., Martin, S.J., Doak, S., Nadeau, N.J.
Wing scale ultrastructure underlying convergent and divergent iridescent colours in mimetic *Heliconius* butterflies.
Journal of the Royal Society Interface

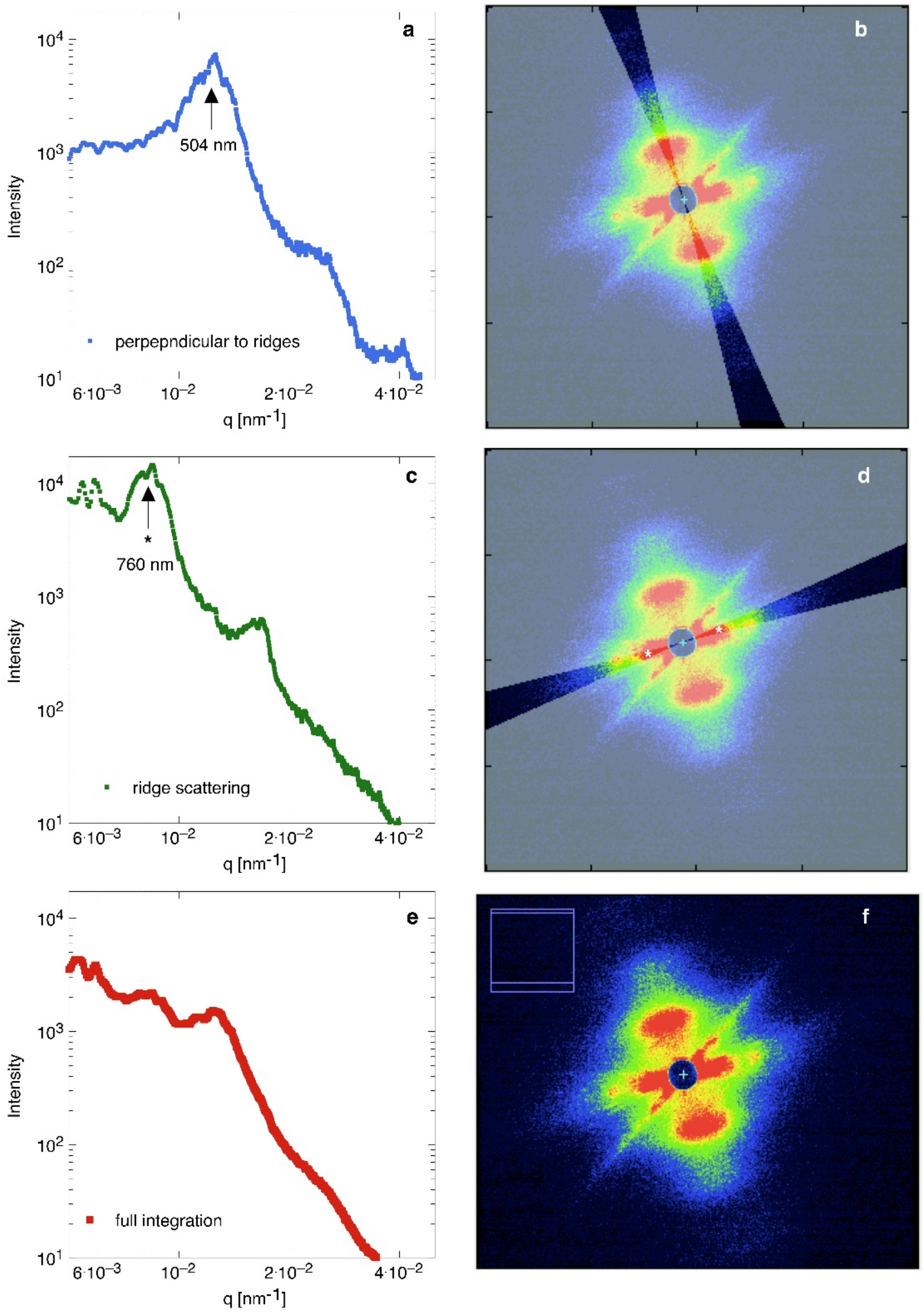

Figure S1 Representative small angle X-ray scattering data for a region of a *H. sara* and the radial integrations of this 2d pattern. Due to the symmetry in the scale architecture we can discern the ridge spacing and cross rib spacing (at 90 ° to the ridges). The radial integration in a is from the cross rib spacing, and involves integrating the region highlighted in b (excluding the contribution from the white-shaded regions). The same masking is applied in d to give the scattering from the ridge spacing in c at q of 0.000829 nm^{-1} (indicated by the arrow and asterisks) and a spacing of 758 nm. The plot in e is the full 360 ° integration of the image in f without any masking of the data, still showing the peak due to the ridge spacing.

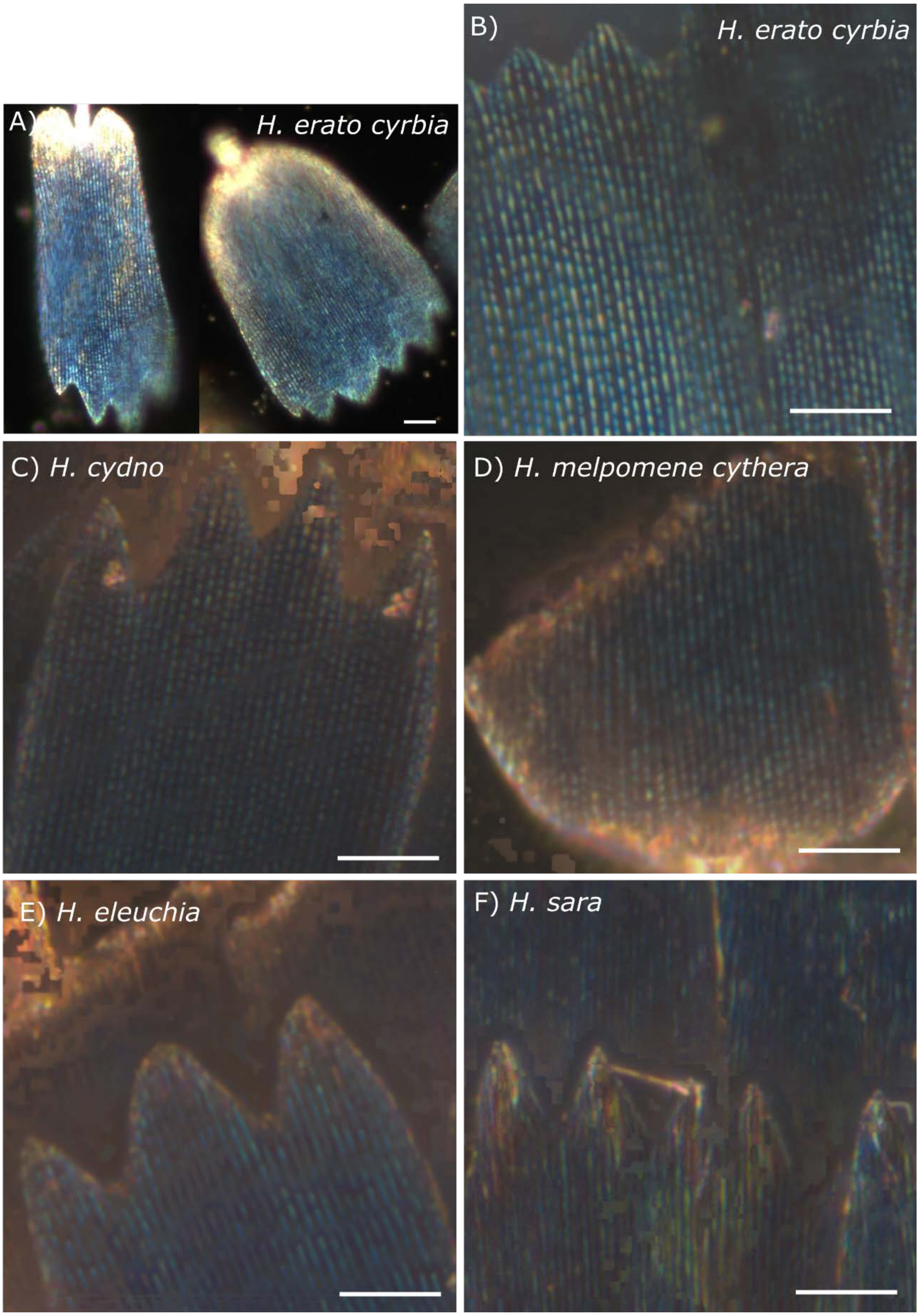

Figure S2 Dark field microscopy images. A) *H. erato cyrbia* individual cover (left) and ground (right) scales, taken with a 50x objective lens. Showing that both scale types reflect blue light. B-F) Extended focus images taken with a 100x objective lens. White bars are 10μm.

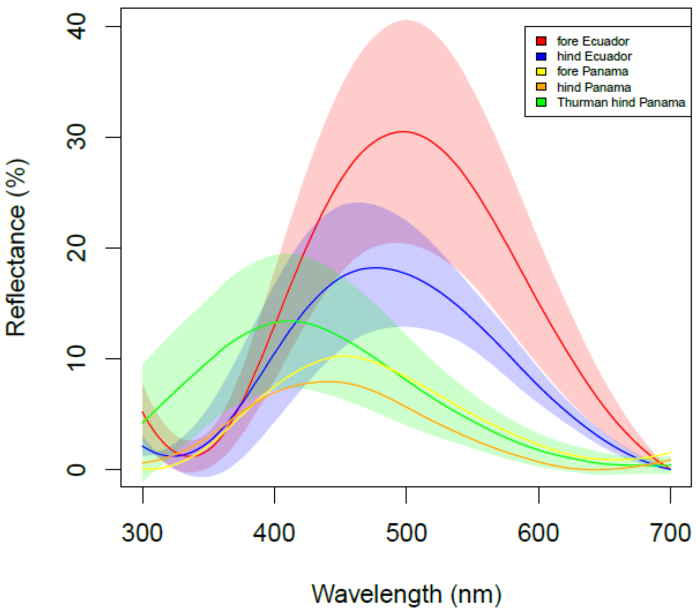

Figure S3 Reflectance spectra for *H. sara* from Panama and Ecuador. Ecuadorian specimens and green-shifted compared to the Panamanian specimens. All reflectance spectra are from the angle of peak reflectance and are shown as the mean and standard deviation of measurements from four individuals. We measured four Ecuadorian *H. sara* (red and blue, fore- and hind-wings respectively) and a single Panamanian specimen (yellow and orange, fore- and hind-wings respectively). These are compared to hind-wing data for four Panamanian *H. sara* from Thurman and Seymoure[1] (green).

1. Thurman TJ, Seymoure BM. A bird's eye view of two mimetic tropical butterflies: coloration matches predator's sensitivity. *J Zool*. 2016;298(3):159-168. doi:10.1111/jzo.12305

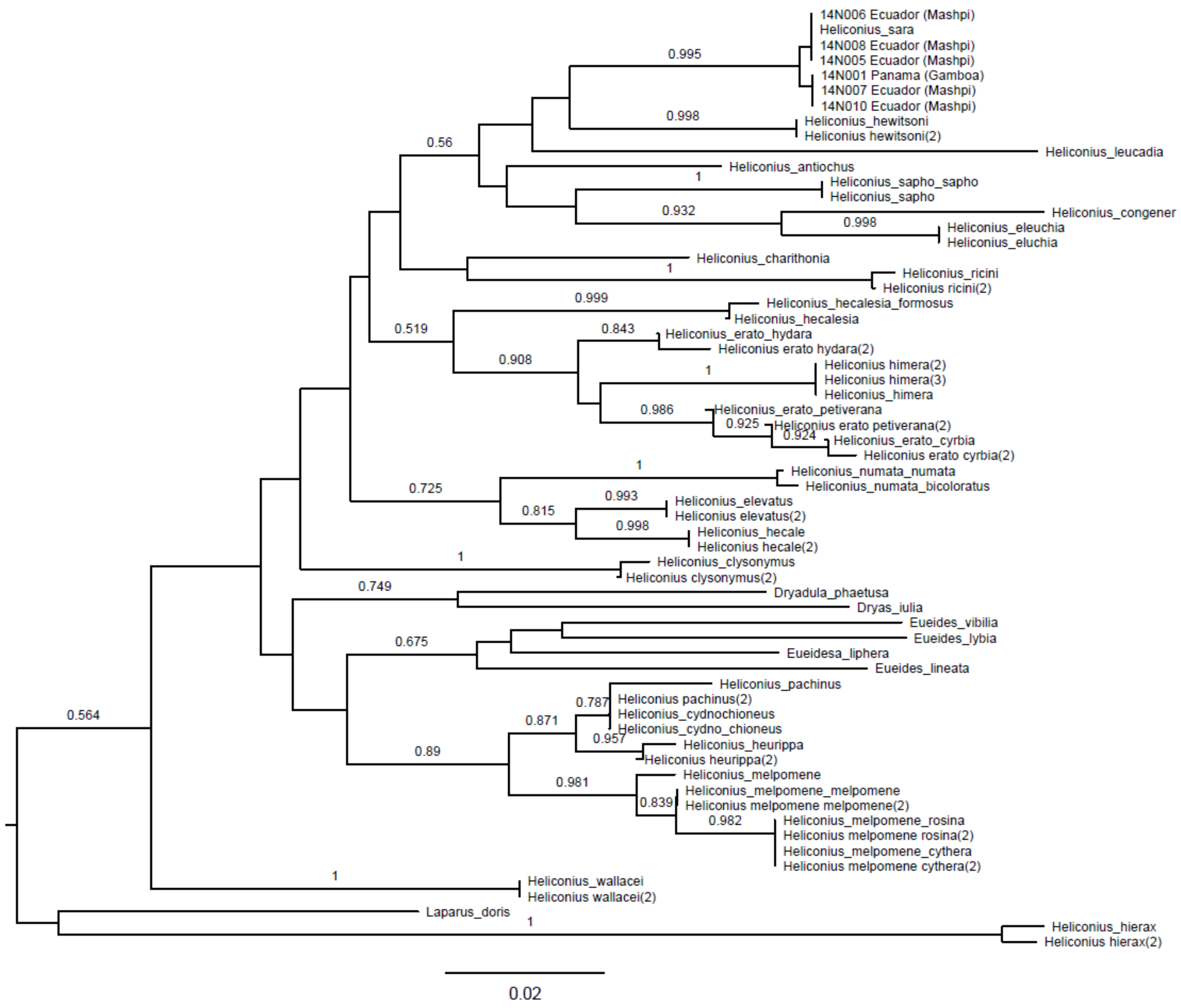

Figure S4 Neighbour joining phylogenetic tree of *Heliconius* species based on 745 bp of mitochondrial *CoI* sequence. The *Heliconius sara* individuals used in this study, from both Panama and Ecuador, group closely with the previously sequenced *H. sara*. Bootstrap support values are shown above branches for clades with >50% support, based on 1000 replicates.

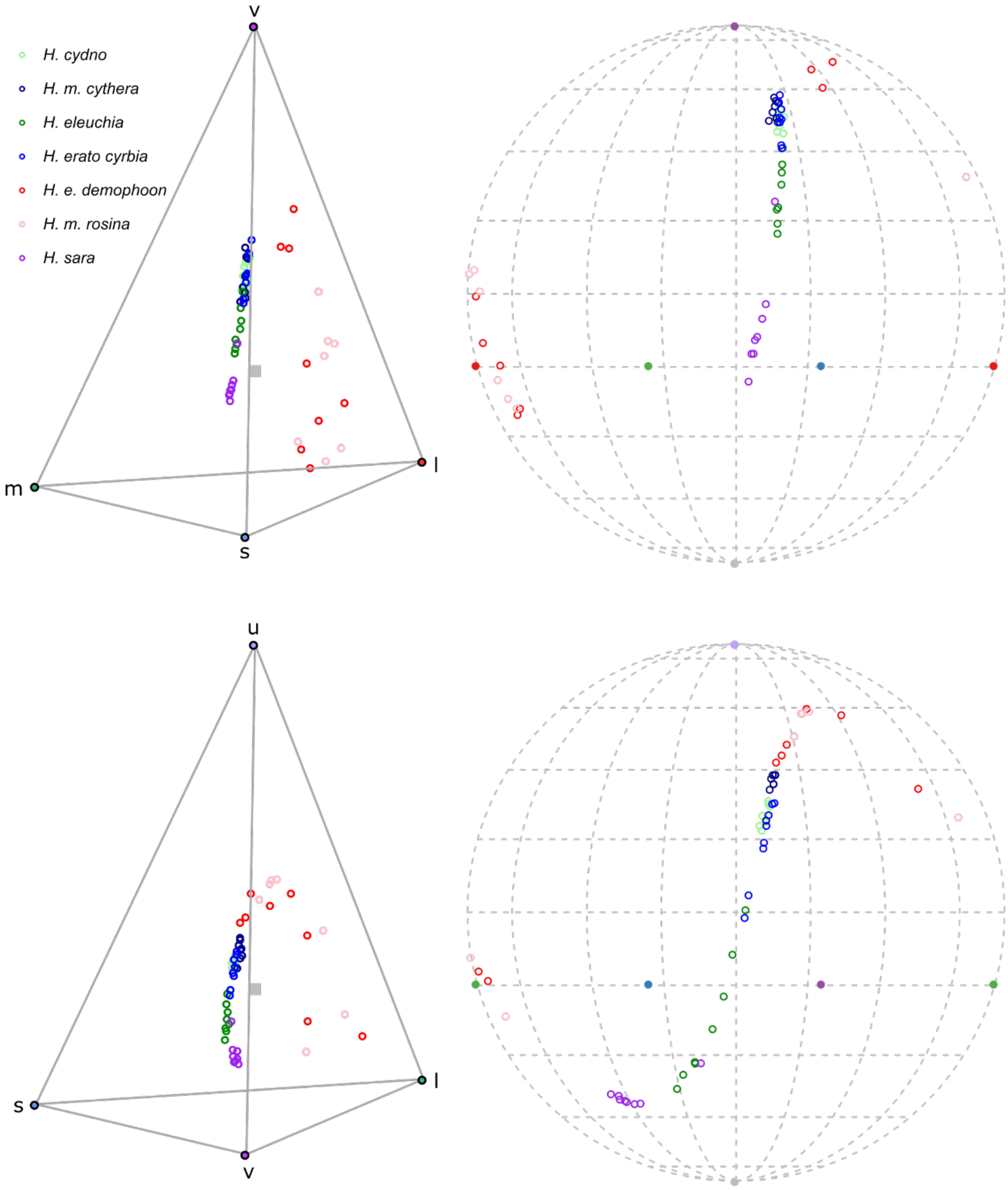

Figure S5. Tetrahedral colour space plots showing the positions of the reflectance spectra from iridescent wing regions (fore and hind, 4 individuals of each species) based on the relative stimulation of the four photoreceptors of birds (top) and *Heliconius* (bottom). The right hand circular plots show these positions projected on the surface of a sphere encompassing the tetrahedron, for easier visualisation.
