# Supplementary material for: Wing scale ultrastructure underlying convergent and divergent iridescent colours in mimetic Heliconius butterflies
Source: J R Soc Interface. 2018 Apr 18;15(141):20170948. doi: 10.1098/rsif.2017.0948 (PMC5938584; doi:10.1098/rsif.2017.0948)
Supplement: Associated data [file rsif20170948supp2.gz › forupload/Butterfly SAXS 1d data.zip!/Butterfly SAXS 1d data/explanation and file information.rtf]

The integrated 1 dimensional small angle x-ray scattering data files used to determine the ridge spacing (see table 1 and figure S4) over a macroscopic region of the wing. The data is scattering intensity as a function of q. The file numbers for the relevant subspecies are listed below.

Heliconius sara SAXS data

7896 
7906
7916 
7926 
7936 
7946 
7956 
7966 
7976 
7986 


Heliconius melpomene rosina SAXS data

2018
2028
2033
2038
2043
2048
2053
2058
2063
2068
2073
2078
2088
2098
2103
2108
2113
2118


Heliconius erato cyrbia SAXS data

9082
9092
9102
9112
9127
9132
9142
9152
9157
9162
9172
9182
9192


Heliconius cydno SAXS data

5421
5431
5451
5462
5471
5481
5484
5491
5501
5511
5521


Heliconius erato demorphon SAXS data

6572
6547
6557
6562
6572
6582
6597
6614
6624
6632


Heliconius eleuchia SAXS data

4311
4320
4330
4340
4350
4360
4370
4380
4390
4400
4410


Heliconius melpomene cythera SAXS data

527
528
529
530
532
534
536
537
